# The prognostic significance of tumor spread through air space in stage I lung adenocarcinoma

**DOI:** 10.1111/1759-7714.14348

**Published:** 2022-02-17

**Authors:** Liling Huang, Le Tang, Liyuan Dai, Yuankai Shi

**Affiliations:** ^1^ Department of Medical Oncology National Cancer Center/National Clinical Research Center for Cancer/Cancer Hospital, Chinese Academy of Medical Sciences & Peking Union Medical College, Beijing Key Laboratory of Clinical Study on Anticancer Molecular Targeted Drugs Beijing China; ^2^ Department of Clinical Laboratory National Cancer Center/National Clinical Research Center for Cancer/Cancer Hospital, Chinese Academy of Medical Sciences & Peking Union Medical College, Beijing Key Laboratory of Clinical Study on Anticancer Molecular Targeted Drugs Beijing China

**Keywords:** lung adenocarcinoma, meta‐analysis, prognosis, spread through air space

## Abstract

**Aim:**

There are still patients of stage I lung adenocarcinoma (ADC) suffering from local or distant recurrence. Herein we conducted a meta‐analysis to investigate the prognostic value of tumor spread through air space (STAS), a new form of invasion pattern, in patients with pathologically confirmed stage I lung ADC.

**Methods:**

Related literature was searched using PubMed, Embase, Cochrane Library, and Web of Science databases from the inception dates to September 4, 2021. Recurrence‐free survival (RFS) and overall survival (OS) were set as primary outcome endpoints. In addition, subgroup analyses on operation mode, edition of the American Joint Committee on Cancer TNM staging, sample size, and research regions were also investigated.

**Results:**

A total of 17 studies involving 9785 patients were included. The presence of STAS was detected in 31.2% of patients and was associated with poor RFS (adjusted hazard ratio [HR] = 1.93, *p* < 0.001) and OS (HR = 2.02, *p* < 0.001). In subgroup analysis on operation mode, the prognostic value of STAS was prominently shown in patients who underwent limited resection (RFS: HR = 3.58, *p* < 0.001; OS: HR = 3.37, *p* < 0.001), while for patients who underwent lobectomy, adverse impact of STAS on RFS was observed (HR = 1.60, *p* = 0.019), but no significant difference was observed on OS (HR = 1.56, *p* = 0.061). The results fluctuated in different regions while other factors did not alter the independent predictive value of STAS.

**Conclusion:**

Tumor STAS should be considered as an adverse prognostic indicator for patients with stage I lung ADC, especially for those under limited resection. More intensive medical care for those patients needs to be investigated in further studies.

## Graphical abstract

Tumor spread through air space (STAS) presents as a new form of invasion pattern in lung adenocarcinoma (ADC), while its prognostic value in stage I lung ADC has not been confirmed. In this meta‐analysis, a total of 17 studies involving 9785 patients of pathologically confirmed stage I lung ADC were included. STAS accounts for 31.2% of the patients and can serve as an indicator for cancer recurrence (adjusted hazard ratio [HR] = 1.93, *p* < 0.001) and worse overall survival (HR = 2.02, *p* < 0.001) in patients with stage I lung ADC, especially for those under limited resection (i.e. sublobular resection). More intensive medical care might be considered for those patients. STAS might help the better stratification and management of patients with stage I lung ADC.

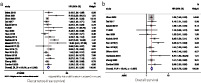

## INTRODUCTION

Lung cancer is one of the most commonly diagnosed cancers and the leading cause of cancer death worldwide according to GLOBOCAN 2020.^1^ Non‐small‐cell lung cancer (NSCLC) accounts for around 85% cases of lung cancer, while lung adenocarcinoma (ADC) serves as a main subtype of NSCLC.^2^, ^3^ Surgical resection serves as the standard of care for patients with stage I NSCLC, whose 5‐year survival rate is roughly 80%.^4^ While there are still patients suffering from local or distant recurrence, identifying those with high risk to recur and who have worse survival is always an unmet clinical need.

The concept of tumor spread through air spaces (STAS) was firstly proposed in the 2015 World Health Organization (WHO) classification of lung tumors, and is defined as spread of tumor cells into air spaces in lung parenchyma beyond the tumor edge.^5^, ^6^ STAS is present in 28.2–37.3% at all stages of lung ADC^7^, ^8^ and is considered as a new form of invasion pattern in NSCLC.^7^ Some retrospective studies have proposed that tumor STAS is valuable in predicting shorter recurrence‐free survival (RFS) and overall survival (OS) of lung ADC,^6^, ^9^, ^10^ while other studies proposed that STAS failed to stratify clinical outcomes such as OS.^11^, ^12^, ^13^ Two meta‐analyses conducted in 2019 proposed the prognostic significance of STAS in NSCLC while those studies only included a small proportion of patients with stage I NSCLC.^8^, ^14^ In the last 2 years, more attention has been paid to the prognostic value of STAS on stage I lung ADC and relevant literature has been published. A pooled analysis of currently available studies is needed to clarify the prognostic significance of STAS on this particular stage. Therefore, we conducted a systemic review and meta‐analysis to investigate whether tumor STAS was closely correlated with recurrence and survival of patients with stage I lung ADC and whether it could help stratify high‐risk patients, who need more intensive medical care.

## MATERIAL AND METHODS

### Search strategy

Four databases, PubMed, Web of Science, Embase, and Cochrane Library, were searched to find relevant prospective or retrospective articles from the inception dates to September 4, 2021. The language was restricted to English. The search strategy was based on the combination of the following terms: “STAS” or “spread through air spaces” and “lung cancer”. Then we specifically focused on articles with analysis outcomes of pathological stage I lung ADC. Furthermore, the reference lists were checked for any relevant articles. The protocol of this study was open on PROSPERO, the International Prospective Register of Systematic Reviews (CRD42021278484).

### Inclusion and exclusion criteria

The inclusion criteria were as follows: (i) retrospective or prospective studies; (ii) studies that enrolled patients who were histologically confirmed as lung ADC; (iii) studies that enrolled patients who were pathologically confirmed as stage I lung ADC; (iv) the association between STAS and survival outcomes (RFS and/or OS) was clarified, and containing corresponding hazard ratio (HR) and 95% confidence interval (95% CI); (v) the language of the studies was limited to English; and (vi) literature research procedure was conducted to September 4, 2021.

The exclusion criteria were as follows: (i) case reports, reviews, meta‐analyses, and conference reports; (ii) duplications; (iii) studies without specific analysis on the prognostic value of tumor STAS on patients with pathologically confirmed stage I lung ADC; (iv) studies that were unable to obtain necessary effect data from the text; and (v) studies that included patients with clinical stage I lung ADC or other histological types.

### Data extraction and quality assessment

Data including first author, year of publication, sample size, number of patients with tumor STAS, country, mean or median age, percentage of male and female patients, edition of the American Joint Committee on Cancer (AJCC) TNM staging which the study was based on,^15^, ^16^ operation modes (lobectomy or limited resection), and outcome (HRs and their 95% CIs for RFS and/or OS) were extracted. Limited resection was defined as sublobular resection, including wedge resection and segmentectomy.^17^

Study screening and quality assessment were performed independently by two reviewers (L.L.H. and L.T.). Study screening and selection were based on inclusion and exclusion criteria by reviewing title, abstract, and full text. Quality assessment was based on the Newcastle‐Ottawa Scale (NOS), which consists of three parts: selection (0–4 points), comparability (0–2 points), and outcome assessment (0–3 points). Disagreement was resolved by discussion or consultation with a third reviewer (L.Y.D.). A study with a NOS score of 6 points or higher was considered to be of high quality.

### Statistical analysis

The effect sizes, namely HRs and corresponding 95% CIs, of tumor STAS on RFS or OS were extracted from the text, tables or supplementary materials provided by each corresponding literature and were pooled to assess the prognostic value of STAS on patients with stage I lung ADC. Cochran's *Q* test and Higgins' *I* ^2^ statistic were performed to analysis the heterogeneity across included studies. If *I* ^2^ were ≤50% and the *p* value was >0.05, the heterogeneity was acceptable.^18^ The nonparametric “trim‐and‐fill” method was used for adjustment and testing the reliability of the findings when high heterogeneity existed.^19^ When no significant heterogeneity was observed, a fixed‐effects model was applied, otherwise we used a random‐effects model. We also conducted sensitivity analysis to assess the influence of each study on the overall result. Begg's funnel plot and Egger's linear regression were performed to assess publication bias.^20^ When the pooled 95% CI did not cross 1 and two‐tailed *p* values <0.05, the difference of two groups was considered of statistical significance and the factor STAS can be served as a prognostic factor. Sensitivity analysis was also conducted by removing each individual study to evaluate the stability of the results. In addition, subgroup analyses on operation mode, histology type, publication year, sample size, and research regions were also investigated. All statistical analyses in this study were conducted by Stata/SE version 15.0 for Windows (Stata Corporation, College Station, TX, USA) and R software (version 4.1.1).

## RESULTS

### Literature search and study characteristics

**FIGURE 1:**
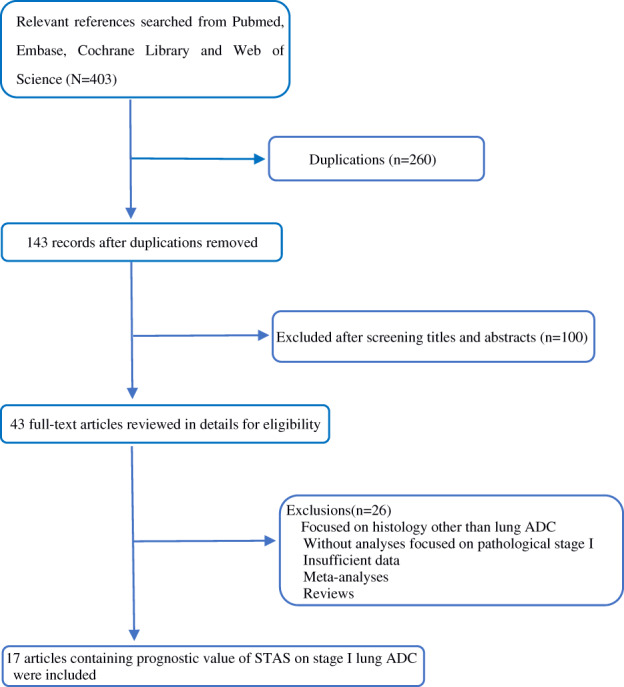
Flow diagram of included studies

A total of 403 records were retrieved from four databases (PubMed, Embase, Cochrane Library, and Web of Science). After removing duplicated publications and screening through titles and abstracts, 43 relevant articles were reviewed in detail for eligibility, and 17 studies published from 2015 to 2021 with a NOS score of 6 or higher met the inclusion criteria and were included in the current meta‐analysis. The selecting flow diagram is summarized in Figure 1.

The detailed characteristics of the incorporated literature are presented in Table 1. Of note study “Toyokawa2 2018”^21^ analyzed patients with limited resection in study “Toyokawa 2018”^22^ and they were from the same population, so the study “Toyokawa2” was only used in subgroup analyses. In the study “Bains 2018”,^23^ no significant association between STAS and RFS was revealed in patients under lobectomy in multivariate analysis, but the related results were not provided, so we only included the subgroup of patients who underwent limited resection (*n* = 352). In total, 9785 patients with stage I lung ADC and a median age of 66 years were included. Among them 3052 patients (31.2%) were detected with status of STAS. In the whole population, effect sizes of correlation between STAS status and RFS were reported in 16 studies, and those between STAS status and OS were available in 11 studies. The region where the studies were conducted included Japan (*n* = 7), China (*n* = 4), Korea (*n* = 3), America (*n* = 2), and Hungary (*n* = 1). The staging of 11 studies was based on the 8th edition of the AJCC TNM staging, the other six studies were based on the 7th edition. Studies published between 2019 and 2021 accounted for two thirds of those included.

**TABLE 1:** Characteristics of studies included in the meta‐analysis

Study	Year	Study design	Country	*N*	STAS (%)	TNM Stage	Analysis type	Age (mean or median)	Sex (proportion of male)	Treatment	AJCC TNM edition	NOS
Bains et al.^23^	2018	R	America	352	126 (36)	stage I	M	NA	36.0%	LIM	8th	7
Chae et al.^38^	2021	R	Korea	115	20 (17.4)	stage IA	M	63.6	47.0%	LIM	8th	6
Chen et al.^33^	2020	R	China	3346	1082 (32.3)	stage I	M	NA	55.4%	LOB or LIM	8th	7
Dai et al.^10^	2017	R	China	383	116 (30.3)	stage IA	M	60	46.5%	LOB or LIM	7th	7
Eguchi et al.^26^	2019	R	America	698	276 (39.5)	stage IA	M	69.5	36.4%	LOB or LIM	8th	7
Yi et al.^12^	2021	R	Korea	109	41 (37.6)	stage I	Uni, M	64.4	35.8%	LOB	8th	6
Han et al.^11^	2020	R	Korea	870	237 (27.2)	stage IA	Uni, M	65	NA	LOB or LIM	8th	7
Hara et al.^39^	2019	R	Japan	245	71 (29)	stage I	M	67	49.8%	LOB	8th	6
Kadota et al.^6^	2015	R	Japan	411	155 (38)	stage I	M	68	39.9%	LOB or LIM	7th	8
Kadota et al.^40^	2019	R	Japan	490	137 (28)	stage I	M	NA	50.6%	LOB or LIM	8th	7
Masai et al.^24^	2017	R	Japan	508	76 (15)	stage I	M	66	48.8%	LIM	7th	8
Ren et al.^41^	2019	R	China	752	225 (29.9)	stage IA	M	NA	NA	LOB or LIM	8th	8
Shiono et al.^42^	2016	R	Japan	318	47 (14.8)	stage I	M	70	46.9%	LOB or LIM	7th	7
Toyokawa et al.^22^	2018	R	Japan	276	153 (55.4)	stage I	M	69	48.6%	LOB or LIM	7th	7
Toyokawa2 et al.^21^	2018	R	Japan	82	31 (37.8)	stage I	M	71	48.8%	LIM	7th	6
Zhong et al.^43^	2020	R	China	620	167 (26.7)	stage I	M	59.6	43.4%	LOB or LIM	8th	7
Zombori et al.^44^	2020	R	Hungary	292	123 (42.1)	stage I	M	62.7	47.3%	LOB or LIM	8th	6

*Abbreviations*: AJCC, American Joint Committee on Cancer; LIM, limited resection; LOB, lobectomy; M, multivariate analysis; NOS, the Newcastle‐Ottawa Scale; R, retrospective; STAS, spread through air space; TNM, tumor node metastasis; Uni, univariate analysis.

### Prognostic significance of STAS in RFS

**FIGURE 2:**
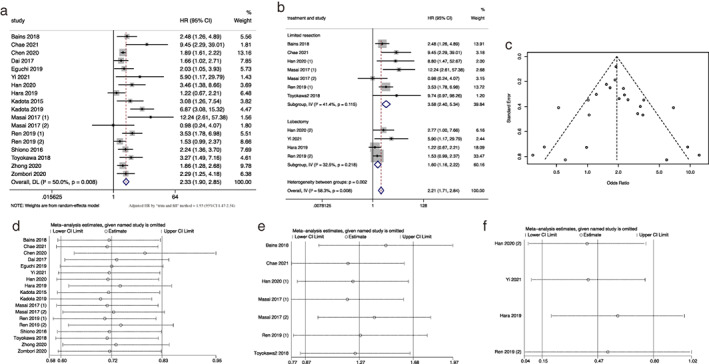
Meta‐analysis of the association between tumor spread through air space and recurrence‐free survival. (a) Forest plot of meta‐analysis in the whole population. (b) Forest plot of meta‐analysis in the subgroup of limited resection and lobectomy. (c) Filled funnel plot using the “trim‐and‐fill” method. Dark circles indicate observed studies, hollow circles in squares indicate missed studies. (d) Sensitivity analysis in the whole population. (e) Sensitivity analysis in the subgroup of limited resection. (f) Sensitivity analysis in subgroup of lobectomy

Sixteen studies reported the association between STAS and RFS. In random effects analysis, STAS is associated with poor RFS of stage I lung ADC patients (HR = 2.33, 95% CI 1.90–2.85, *p* < 0.001; Figure 2a), while heterogeneity (*I* ^2^ = 50.0%, *p* = 0.008) and publication bias (*p* value of Egger's test = 0.014) were revealed across these studies. The nonparametric “trim and fill” method was applied to detect the stability of our results. The adjusted HR was 1.93 for RFS (95% CI 1.47–2.54, *p* < 0.001; Figure 2c), which still confirmed the prognostic value of STAS on RFS of patients with stage I lung ADC.

We conducted a subgroup analysis based on the operation method (limited resection vs. lobectomy). The association between STAS and RFS of patients under limited resection and lobectomy were presented in six and four studies, respectively. Among them, the study of Masai et al.^24^ has separate results of local recurrence [Masai 2017 (1)] and distant recurrence [Masai 2017 (2)].

In our subgroup analysis, 1357 patients underwent limited resection (numbers of study = 6) and 1636 patients underwent lobectomy (numbers of study = 4). As shown in Figure 2b, patients with STAS had a poor RFS under both limited resection (HR = 3.58, 95% CI 2.40–5.34, *p* < 0.001) and lobectomy (HR = 1.60, 95% CI 1.16–2.22, *p* = 0.019), and no significant heterogeneity and publication bias were observed in either subgroup (limited resection: *p* of heterogeneity = 0.115, *p* of Egger's test = 0.233; lobectomy: *p* of heterogeneity = 0.218, *p* of Egger's test = 0.145). As shown above, the adverse impact of STAS on RFS was stronger in the subgroup of limited resection. Figure 2d–f shows the results of sensitivity analysis in the whole group and the subgroups of limited resection and lobectomy, indicating the stability of the pooled results.

### Prognostic significance of STAS in OS

**FIGURE 3:**
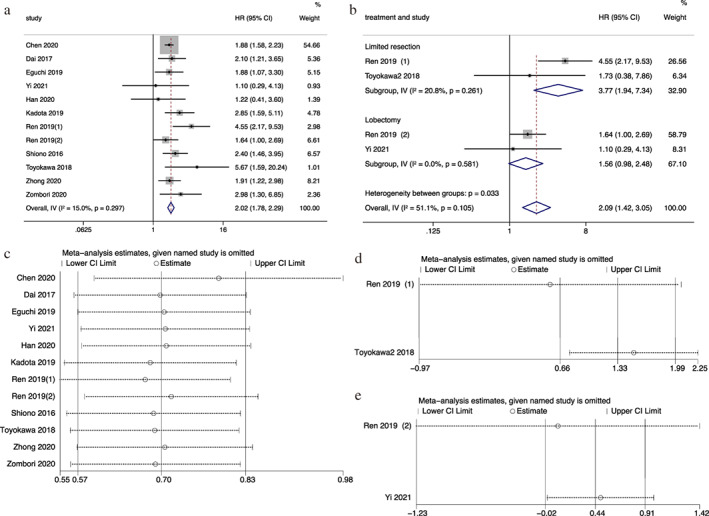
Meta‐analysis of the association between tumor spread through air space and overall survival. (a) Forest plot of meta‐analysis in the whole population. (b) Forest plot of meta‐analysis in the subgroup of limited resection and lobectomy. (c) Sensitivity analysis in the whole population. (d) Sensitivity analysis in the subgroup of limited resection. (e) Sensitivity analysis in the subgroup of lobectomy

Eleven studies reported the association between STAS and OS of patients with stage I lung ADC. Patients with STAS were shown to have shorter OS (HR = 2.02, 95% CI 1.78–2.29, *p* < 0.001; Figure 3a), besides the heterogeneity (*I* ^2^ = 15.0%, *p* = 0.297) and publication bias (*p* of Egger's test = 0.216) among these studies was acceptable. Our meta‐analysis showed that STAS is likely to be a adverse prognostic indicator for OS. In the subgroup analysis of operation mode, patients with STAS had an inferior OS under limited resection (numbers of study = 2, HR = 3.77, 95% CI 1.94–7.34, *p* < 0.001), while no significant statistical difference was observed in patients under lobectomy (numbers of study = 2, HR = 1.56, 95% CI 0.98–2.48, *p* = 0.061) (Fig. 3b). Excluding any of studies did not change the pooled HR and corresponding 95% CI qualitatively in the sensitivity analysis (Fig. 3c–e).

### Other subgroup analyses of STAS in survival outcome

The detailed results of the subgroup analyses of RFS and OS are summarized in Tables 2 and 3. Although the definition of stage I in the 7th and 8th editions of AJCC TNM staging differs, the prognostic value of STAS in both editions of AJCC TNM staging has been confirmed. Patients with STAS were associated with poor RFS in both the 7th (HR = 2.43, 95% CI 1.58–3.72, *p* < 0.001) and 8th (HR = 2.32, 95% CI 1.81–2.96, *p* < 0.001) editions. However, heterogeneity (*I* ^2^ = 55.5%, *p* = 0.01) and publication bias (*p* of Egger's test = 0.039) were observed in the subgroup of the 8th edition. STAS was also related to poor OS in both the 7th (HR = 2.43, 95% CI 1.70–3.46, *p* < 0.001) and 8th (HR = 1.96, 95% CI 1.71–2.25, *p* < 0.001) editions, with no significant heterogeneity and publication bias in both subgroups. The results in different research regions showed different HRs of STAS for RFS and OS, while almost all confirmed the value of STAS except for OS analysis in Korea (*p* = 0.715). Additionally, sample size (≤400 or >400) did not alter the independent predictive value of STAS on either RFS or OS in subgroup analysis. STAS is likely to be a solid adverse prognostic factor for survival outcome.

**TABLE 2:** Subgroup analyses of the association between STAS and RFS

		Test of association			Test of heterogeneity		Egger's test		Begg's test	
Variable	Studies	HR	95%CI	*p* value	*I* ^2^	*p* value	*t* (Bias)	*p* value	*z*	*p* value
Total	16	2.33	1.90–2.85	<0.001	50.0%	0.008	2.77	0.014	3.11	0.002
Treatment										
Limited resection	6	3.58	2.40–5.34	<0.001	41.4%	0.115	1.36	0.233	0.6	0.548
Lobectomy	4	1.6	1.16–2.22	0.019	32.5%	0.218	2.33	0.145	1.02	0.308
AJCC TNM										
7th	5	2.43	1.58–3.72	<0.001	42.9%	0.119	1.48	0.334	1.13	0.260
8th	11	2.32	1.81–2.96	<0.001	55.5%	0.01	1.43	0.039	2.81	0.005
Research region										
Korea	3	4.86	2.43–9.74	<0.001	0.0%	0.49	1.5	0.394	0.00	1.000
China	4	1.88	1.61–2.19	<0.001	8.9%	0.356	0.33	0.766	−0.24	1.000
America	2	2.24	1.39–3.59	0.001	0.0%	0.679	–	–	0.00	1.000
Japan	6	2.82	1.64–4.86	<0.001	67.2%	0.006	0.96	0.382	0.60	0.548
Hungary	1	2.29	1.25–4.18	0.007	–	–	–	–	–	–
Sample size										
≤400	7	2.25	1.64–3.09	<0.001	39.5%	0.116	2.82	0.024	2.60	0.009
>400	9	2.43	1.82–3.25	<0.001	59.6%	0.008	1.34	0.085	1.79	0.074

*Abbreviations*: AJCC, American Joint Committee on Cancer; RFS, recurrence‐free survival; STAS, spread through air space; TNM, tumor, node, metastasis.

**TABLE 3:** Subgroup analyses of the association between STAS and OS

		Test of association			Test of heterogeneity		Egger's test		Begg's test	
Variable	Studies	HR	95%CI	*p* value	*I* ^2^	*p* value	*t* (Bias)	*p* value	*z*	*p* value
Total	11	2.02	1.78–2.29	<0.001	15.0%	0.297	1.32	0.216	1.03	0.304
Treatment										
Limited resection	2	3.77	1.94–7.34	<0.001	20.8%	0.261	–	–	0.00	1.000
Lobectomy	2	1.56	0.98–2.48	0.061	0.0%	0.581	–	–	0.00	1.000
AJCC TNM										
7th	3	2.43	1.70–3.46	<0.001	18.1%	0.282	2.29	0.242	0.00	1.000
8th	8	1.96	1.71–2.25	<0.001	0.0%	0.372	0.41	0.554	−0.10	1.000
Research region										
Korea	2	1.17	0.51–2.70	0.715	0.0%	0.907	–	–	0.00	1.000
China	4	1.94	1.68–2.24	<0.001	30.6%	0.217	1.06	0.365	1.22	0.221
America	1	1.88	1.07–3.30	0.028	–	–	–	–	–	–
Japan	3	2.75	1.91–3.96	<0.001	0.0%	0.462	6.14	0.103	1.04	0.296
Hungary	1	2.98	1.30–6.85	0.01	–	–	–	–	–	–
Sample size										
≤400	5	2.39	1.74–3.28	<0.001	0.0%	0.466	0.38	0.79	−0.24	1.000
>400	6	1.95	1.698–2.25	<0.001	25.60%	0.234	0.61	0.496	0.60	0.548

*Abbreviations*: AJCC, American Joint Committee on Cancer; OS, overall survival; STAS, spread through air space; TNM, tumor, node, metastasis.

## DISCUSSION

This meta‐analysis of 9785 patients with stage I lung ADC revealed that STAS is a significant adverse prognostic indicator for recurrence (adjusted HR = 1.93, 95% CI 1.47–2.54, *p* < 0.001) and overall survival (HR = 2.02, 95% CI 1.78–2.29, *p* < 0.001) regardless of the extent of resection. Moreover, the prognostic value of STAS for recurrence is prominently shown in those who underwent limited resection (RFS: HR = 3.58, 95% CI 2.40–5.34, *p* < 0.001; OS: HR = 3.77, 95% CI 1.94–7.34, *p* < 0.001) with low heterogeneity, and the results are consisted with previous studies.^24^, ^25^, ^26^ In patients under lobectomy, STAS was shown to be related to a shorter RFS, while no significant difference was observed in OS analysis (*p* = 0.061), which may due to the limited number in this subgroup (*n* = 743). The results fluctuated in different regions while other factors, such as histology, edition of AJCC TNM staging, and sample size, did not alter the independent predictive value of STAS in subgroup analysis.

In general, surgery provides the best chance of cure for patients with stage I NSCLC.^27^ According to the latest National Comprehensive Cancer Network® (NCCN®) guidelines, limited resection is appropriate in selected patients under the following criteria: (i) unable to undertake lobectomy for poor pulmonary reserve or other major comorbidity; (ii) peripheral nodule ≤2 cm with histology of pure ADC in situ or nodule has ≥50% ground‐glass appearance on computed tomography or takes ≥400 days to get a double size under radiologic surveillance. In a meta‐analysis, no significant survival difference was observed in patients with stage I lung cancer under limited resection or lobectomy,^17^ but our analysis revealed that patients with STAS‐positive stage I lung ADC were associated with higher risk of recurrence and worse OS, especially those under limited resection.

Yasuhiro et al. conducted a study on adjuvant chemotherapy for high‐risk stage I NSCLC which revealed that age >70 years, invasive component size >2 cm, visceral pleural invasion, and vascular or lymphatic invasion were independent factors for RFS, and adjuvant chemotherapy for high‐risk stage I patients prolonged RFS and OS significantly.^28^ In previous literature, STAS was found to be associated with aggressive clinicopathologic characteristics in surgically resected lung ADC.^29^ In imaging finding, STAS is associated with higher pathological stage, a larger tumor diameter, higher presence of solid component, and vascular convergence.^30^, ^31^ In terms of pathology finding, STAS was strongly linked to the presence of lymphovascular invasion and high‐grade morphologic patterns, including larger nuclear size, increased mitotic count, and high Ki‐67 labeling index, which suggests that STAS can serve as a marker for tumor proliferation.^29^, ^32^ Chen et al. carried out a multi‐institutional study to investigate whether stage I ADC with STAS can benefit from adjuvant chemotherapy. The results showed that for patients with STAS‐positive stage IB lung ADC and those with STAS‐positive stage IA lung ADC who underwent limited resection, adjuvant chemotherapy could bring about better survival.^33^ Therefore, for patients with stage I lung ADC who received limited surgical resection and were presented with tumor STAS, more intensive medical care, such as extra lobectomy (when STAS was diagnosised on frozen section during operation) or postoperative treatment, needs to be discussed in the further clinical decision‐making process. Moreover, a predictive model of STAS has been developing based on radiomics with machine learning, which can help clinicians to identify possible STAS before operation.^34^, ^35^

STAS can be further graded into STAS I and STAS II based on the distance from the edge of tumor to the presence of STAS (STAS I: distance <2500 μm; STAS II: distance ≥2500 μm). Another study reported that in 1869 patients with stage IA non‐mucinous ADC, STAS I accounted for 24.4% and STAS II accounted for 16.5% of those patients. STAS II was an adverse prognostic indicator but not STAS I.^11^ Of note, some studies proposed that STAS might be an ex vivo artifact caused by spreading through a knife surface.^36^, ^37^ Importantly, surgical manipulation and slide preparation need to be better standardized to avoid false reporting of tumor STAS.

Limitations remain in this study. First, publication bias is unavoidable since studies with negative results might not be published, but we have included potential confounding factors into subgroup analysis to minimize the bias. Second, we only included study with the histology of lung ADC since we found few studies on histology other than lung ADC. More investigation on the prognostic value of STAS on lung squamous cell carcinoma or other subtypes of NSCLC is needed.

## CONCLUSION

In this meta‐analysis, tumor STAS was confirmed as an independent adverse prognostic indicator for patients with pathological stage I lung ADC, especially for those under limited resection. More intensive medical care for these patients needs to be investigated in further study.

## CONFLICT OF INTEREST

The authors have no conflicts of interest to declare.

## AUTHOR CONTRIBUTIONS

Y.K.S. contributed to the conception and design of the study. L.L.H., L.T., and L.Y.D. collected and summarized the data. L.L.H. conducted the statistical analysis and drafted the manuscript. All authors revised the manuscript critically for important intellectual content. The final approval of the manuscript was obtained from all authors.
